# Timing of Decompressive Craniectomy for Malignant Middle Cerebral Artery Infarction: A Single-Center Analysis

**DOI:** 10.3390/medicina55020031

**Published:** 2019-01-30

**Authors:** Mustafa Kilic, Devrimsel Harika Ertem, Burak Ozdemir

**Affiliations:** 1 Department of Neurosurgery, Şişli Hamidiye Etfal Training and Research Hospital, University of Health Sciences, Istanbul 34371, Turkey; kilicnrs@gmail.com (M.K.); drburakozdemir37@gmail.com (B.O.); 2 Department of Neurology, Şişli Hamidiye Etfal Training and Research Hospital, University of Health Sciences, Istanbul 34371, Turkey

**Keywords:** acute cerebral ischemia, decompressive craniectomy, malignant middle cerebral artery infarction, outcomes

## Abstract

*Background and aim*: Malignant middle cerebral artery infarction (MMCAI) usually leads to brain edema that may result in transtentorial herniation and brainstem compression. The prognosis of MMCAI is generally poor. The aim of this study was to discuss our experience with surgical decompression for MMCAI, and determine the association between timing of craniectomy and neurological outcomes. *Methods*: We identified consecutive patients diagnosed with MMCAI who underwent decompressive craniectomy (DC). Clinical and demographic data were obtained from electronic medical records, including: age, sex, preoperative Glasgow Coma Scale (GCS) score, surgery timing, postoperative GCS scores, and modified Rankin Scale (mRS) scores. *Results*: This study included 27 stroke patients (aged 38–80 years) operated within 72 h of the onset of neurological symptoms. Sixteen, five, and six patients underwent DC within 24 h, between 24 and 48 h, and after 48 h after onset of symptoms, respectively. Five patients died after the surgery. Patients who underwent DC within 24 h and 24–48 h had better mean GCS scores than those who underwent DC after 48 h (*p* = 0.000, *p* = 0.015). In addition, patients who underwent DC within 24 h had better mean postoperative mRS scores (*p* = 0.000) than other patients. Patients older than 60 years had significantly lower GCS scores (*p* = 0.027) and higher mRS scores (*p* = 0.033) than younger patients. *Conclusion*: Our findings support that DC had satisfying outcomes in patients who underwent DC within 24 h. Older age and lower Glasgow Coma Scale scores among DC patients with MMCAI are associated with high morbidity and mortality.

## 1. Introduction

Malignant middle cerebral artery infarction (MMCAI) is the term used to describe rapid neurological deterioration due to the effects of space-occupying cerebral edema, between 24 h and 72 h following acute middle cerebral artery ischemic stroke [1]. The prognosis of MMCAI is generally poor. In a prospective case series, 78% died from herniation despite maximum conservative treatment [2]. Decompressive craniectomy (DC) is one of the surgical options to treat brain edema, and lessens the risk of brain herniations and death. DC is a surgical treatment strategy that may address the vicious cycle between infraction and cerebral edema, which causes more occlusion due to insufficient space and high intracranial pressure. It has been reported that early DC is related to lower neurological deficits, and an earlier return to activities of daily life [3]. DC includes the temporary removal of some bone fragments (e.g., frontal, temporal, and parietal), followed by a duraplasty procedure to provide space for the brain. In the 1890s, Annandale first described the procedure for decompression. In 1905, Kocher and Cushing suggested performing DC to decrease high intracranial pressure, but the procedure was not performed due to aesthetic concerns [4]. It has been suggested that the prognostic factors of DC patients with MMCAI vary, including age of patient, brain herniation degree, and preoperative neurological status. Patients younger than 60 years undergoing surgery as early as possible have more satisfactory results [5]. A prospective, randomized trial provided evidence that DC is a life-saving procedure, increasing the chances of survival from 29% to 78% [3]. The timing of decompressive surgery and appropriate patient selection based on age require careful clinical judgement. However, there are a limited number of studies evaluating the relationship between the timing of surgery and its outcomes. It has been reported that the timing of intervention was not associated with in-hospital mortality, but delayed surgery was associated with a poor outcome [6]. The aim of this study was to discuss our experience with DC for MMCAI, and determine the association between craniectomy timing and neurological outcomes.

## 2. Materials and Methods

### 2.1. Participants

This retrospective, cross-sectional study was approved by the ethics committee of the University of Health Sciences, Sisli Hamidiye Etfal Training and Research Hospital, and was performed in accordance with the Declaration of Helsinki. We reviewed the records of 27 patients who had undergone surgical intervention for MMCAI and who were admitted within 72 h of acute ischemic infarction during a 24-month period from 2015 to 2017 in our neurosurgery department.

Relevant clinical and demographic data were obtained from the electronic medical records, including: age, sex, preoperative Glasgow Coma Scale (GCS) scores, surgery timing, infarction side, hemiparesis or hemiplegia side, early-term postoperative GCS scores, and modified Rankin Scale (mRS) score. 

Patients were grouped based on surgery timing. The first, second, and third groups included patients who underwent surgery within 24 h, between 24 and 48 h, and 48 h after the onset of symptoms, respectively. During follow-up, the mRS scores were recorded.

### 2.2. Surgical Procedure

**Figure 1:**
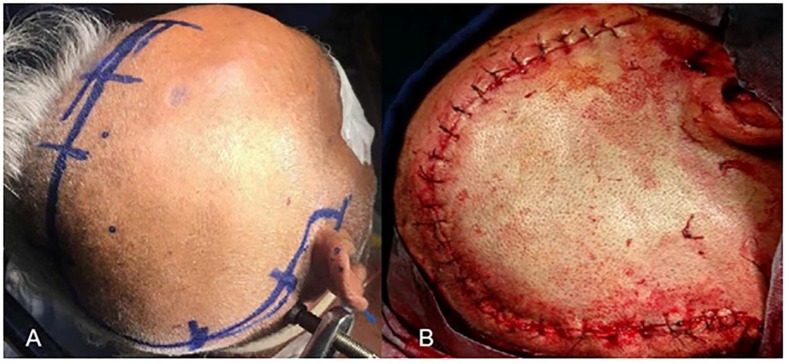
Surgical procedure of decompressive craniectomy (DC) for malignant middle cerebral artery infarction.

All procedures were performed in our neurosurgery department. The surgical procedure included a wide fronto-temporo-parietal skin flap (Figure 1A). The eligibility criteria for patients undergoing decompressive craniectomy in the current study included: meeting clinical and radiological criteria of MMCAI, radiologically-proven diffuse edema, at least a 0.5 cm midline shift, and rapid neurological deterioration despite medical treatment. Patients with multiple and bilateral infarctions and patients with massive intraventricular and/or intracerebral hemorrhage were excluded from DC. The burr holes were opened and cut towards each other using a drill, followed by removing a 15 cm × 15 cm free bone flap on the infarction side. The dura was incised, followed by duraplasty using a dura graft that was usually excised from the galea. No lobectomy or intracortical procedure was performed. After the duraplasty, the temporal muscle was sutured loosely above the dura. All anatomical structures were closed properly, and the procedures were completed without any perioperative complications (Figure 1B). The cranioplasty was performed approximately 12 weeks after DC.

### 2.3. Statistical Analysis

Data were presented as the mean ± standard deviation (SD), whereas categorical variables were presented as numbers and percentages. Wilcoxon signed-rank tests and Pearson’s chi-square tests were used to compare variables. Correlations among the scales were evaluated using the Spearman’s rank correlation analysis. All statistical analyses were performed using the Statistical Package for the Social Sciences (SPSS) 23.0 (SPSS Inc., Chicago, IL, USA, 2005), with a level of significance set at *p* < 0.05.

## 3. Results

In total, 27 patients (18 male and 9 female) underwent DC for MMCAI. The mean age of the patients was 54.4 ± 13.1 years (range 38–80 years). Twelve patients were 60 years or older, whereas 15 patients were younger than 60 years. Seventeen and 10 patients had right- and left-sided infarctions, respectively. Thirteen patients had dominant hemisphere lesions, and 14 had nondominant hemisphere lesions. Twenty-one patients (78%) had at least one of the following: coronary artery disease, hypertension, or diabetes mellitus. Coronary artery disease (*n* = 11) and hypertension (*n* = 9) were the two most prevalent stroke etiologies. Before surgery, the mean GCS score of all patients was 10.03 ± 2.03 (range 7–13). Five patients died postoperatively in the neurosurgical intensive care unit (NICU). Six patients had an mRS score of 4–5 during discharge, whereas the other patients had mRS scores of 0–3. 

Table 1 shows the preoperative and postoperative mean GCS and mRS scores, and the number of patients who died after DC among groups who underwent surgery within 24 h, between 24 and 48 h, and 48 h after the onset of neurological deterioration, respectively. Patients who underwent DC within 24 h (Group 1) and 24–48 h (Group 2) had better mean GCS scores than those who underwent DC after 48 h (Group 3) (*p* = 0.000, *p* = 0.015). Group 1 had better mean postoperative mRS scores (*p* = 0.000). However, no statistically significant difference was found in the postoperative mean mRS scores among Groups 2 and 3.

Patients with preoperative GCS scores between 10 and 14 were discharged with a better mRS (mean = 2) score, whereas those with GCS scores ≤9 had a higher incidence of poor outcomes and were discharged with a higher mRS (mean = 5.5) score. 

Patients older than 60 years had significantly lower GCS scores (*p* = 0.027) and higher mRS scores (*p* = 0.033) compared to younger patients. Gender, hemisphere dominancy, and infarction side were not associated with GCS and mRS scores, nor associated with mortality (Table 2).

### Illustrative Cases

**Figure 2:**
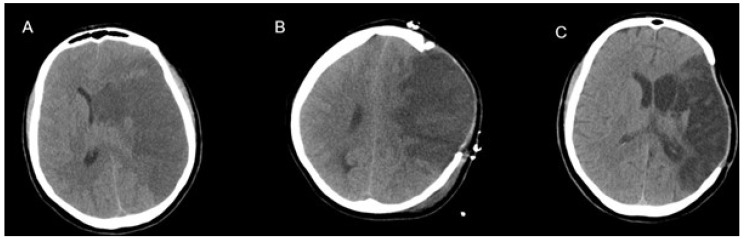
Computed tomography (CT) scans of a 37-year-old female patient with left-sided MMCAI (malignant middle cerebral artery infarction) (**A**). Postoperative 1st day CT (**B**). Postoperative third week CT showed no midline shift (**C**).

Figure 2 demonstrates a 37-year-old female patient with left-sided MMCAI (A). Preoperative GCS score was 11. Decompressive surgery was performed within 24 h, and a computed tomography (CT) scan was taken 1 day after surgery (B). She had a GCS score of 12 in the early postoperative period. A postoperative CT in the third week showed excellent results without a midline shift (C), and the patient had a GCS score of 15.

**Figure 3:**
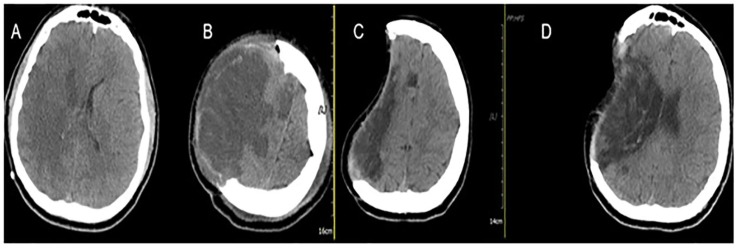
Computed tomography (CT) scans of a 69-year-old male patient with right-sided MMCAI (**A**). Postoperative CT shows the wide craniectomy region (**B**). After two months, CT showed favorable results without midline shift (**C**,**D**).

Figure 3 demonstrates a 69-year-old male patient with right-sided MMCAI (A). Preoperative GCS score was 12, and the decompression surgery was performed within 24 h. Postoperative CT shows the wide craniectomy region (B). After two months, CT showed favorable results without midline shift (C,D), and the patient’s GCS score was 15.

## 4. Discussion

Acute ischemia involving the entire region of the middle cerebral artery of a hemisphere may cause space-occupying cerebral edema, leading to rapid neurological deterioration with symptoms such as hemiparesis, gaze deviation following by headache, vomiting, and changes in level of consciousness [7,8]. As a result of infarction, brain edema and brain herniation can result in an 80% mortality rate. Surgical intervention for MMCAI should be considered when CT imaging indicates an ischemic lesion occupying >50% of the middle cerebral artery territory [9]. In patients with MMCAI, clinical worsening often occurs in the first 24–48 h. The timing of decompressive surgery is very important to prevent permanent neurological deterioration. This study aimed to demonstrate the clinical outcomes of DC for MMCAI to assess the importance of surgery timing. Our results demonstrated that older age and lower GSC scores were related to higher morbidity. Although the operation methods have not been clearly established, there are limited studies investigating the outcome of procedure types. Decompressive surgery that includes only bone removal reduces intracranial pressure by approximately 15%, but this reduction can be increased up to 70% if the dura is opened [9]. In our study, all patients underwent surgery with duraplasty. Many authors suggest the removal of at least 14 cm × 10 cm of the fronto-temporo-parietal bone flap [10]. In our study, we removed a 15 cm × 15 cm bone flap. Although a craniectomy measuring 8–10 cm can provide sufficient space for an additional 25 mL of volume, removing >10 cm of bone provides 80–90 mL of additional volume. According to our results, gender, hemisphere dominancy, and infarction side were not associated with the GCS and mRS scores and mortality. In the literature, some studies suggested surgical treatment in the non-dominant hemisphere for improved outcomes. However, other studies showed that no difference was found in outcomes dependent on the hemisphere [11,12,13]. Our findings support that DC in the dominant hemisphere was not associated with worse outcomes. 

Two randomized, controlled trials of early decompressive craniectomy in MMCAI reported that early DC (performed within 24 h after onset of symptoms) significantly reduced mortality, and improved outcomes 6 and 12 months after stroke [12,14]. In the DECIMAL study, the outcomes of surgery and conservative treatment were compared four weeks after surgery. The surgery group had a 16% mortality rate and the conservative group had a 33% mortality rate [12]. The mortality rate was found to be 18% in this study. In the HAMLET study, another randomized trial, it has been reported that surgical decompression reduces poor outcomes in patients with infarctions treated within 48 h of stroke onset. Moreover, it has been suggested that there is no evidence that DC improves functional outcome when it is delayed for up to 96 h after stroke onset [11]. According to our experience, performing an early surgical operation in patients with space-occupying ischemic infarctions may contribute to reduced case fatalities and better outcomes.

In our retrospective study, 60% of patients had a statistically significant reduced mRS score after DC. Similar to our results, a previous study showed that DC provided favorable mRS scores (0–3) in 22% of patients, compared with the conservative treatment group [15]. Another important prognostic factor for patients with MMCAI is postoperative GCS scores. Some studies demonstrated that postoperative GCS scores can affect follow-up outcomes [2,12,16]. In the current study, better mRS scores were found in patients with high postoperative GCS scores.

Because of the risks of this procedure, the exact criteria of DC for patients with MMCAI still remain unclear. DC is associated with preventing brain damage by reducing intracranial pressure and cerebral herniation, though it is not always associated with a good clinical course [17]. Surgical complications such as insufficient decompression, infections, hydrocephalus, hemorrhage, and subdural collections, can worsen the clinical outcome of these patients [18,19]. Recently, seizures and epilepsy after DC for MMCAI was demonstrated by Brondani et al. [20]. They found that seizures occurred in 61.1% of 36 patients with MMCAI after the surgical procedure. In this retrospective study, researchers analyzed the incidence of, and the risk factors for, seizure or epilepsy development in these patients. They reported that although epilepsy was very frequent, they did not detect any particular variable as a risk factor for the development of epilepsy. We did not observe any convulsive seizures (partial or generalized tonic–clonic seizures) following the DC; however, the diagnosis of nonconvulsive status epilepticus cannot be eliminated. The importance of nonconvulsive status epilepticus is that nonconvulsive/subclinical seizures may contribute to lower postoperative GCS scores. Further electrophysiological studies are needed to confirm the diagnosis of nonconvulsive status epilepticus to highlight the contribution of nonconvulsive seizures to clinical deterioration.

This study had some limitations. First, because it was a retrospective study, only eligible data in charts were evaluated. Second, the study was performed at a single center. However, patients were grouped according to the onset of their symptoms and timing of operation, so that their clinical survey was compared with their neurological status.

## 5. Conclusions 

In conclusion, our findings support the evidence that DC for MMCAI can decrease morbidity and mortality rates. Early decompressive surgery reduced mortality in those patients younger than 60 years, and contributed to improved functional outcomes (mRS score ≤ 3). The timing of decompression, age, and preoperative and postoperative neurological status are prognostic factors that affect the clinical outcomes of patients.

## Figures and Tables

**Table 1:** The preoperative and postoperative clinical outcomes of patients after DC due to timing.

	*n*	Timing of Surgery	Number of Deaths	Mean GCS Scores		*p* Value	Mean mRS Scores		*p* Value
				Preop.	Postop.		Preop.	Postop.	
Group 1	16	<24 h	2	8.7	14.5	0.000	4.2	2.3	0.000
Group 2	5	24–48 h	1	7.02	9	0.015	4.8	4.5	0.823
Group 3	6	>48 h	2	11.38	12.75	0.330	5.6	5.5	0.970

GCS—Glasgow Coma Scale, mRS—modified Rankin Scale.

**Table 2:** The Glasgow Coma Scale (GCS) and modified Rankin Scale (mRS) scores and number of deaths after DC.

		*n*	GCS Scores		mRS		Deaths	
			Mean ± SD	*p* Value	Mean ± SD	*p* Value	*n*	*p* Value
Age	<60	15	13.42 ± 1.89	0.027	2.87 ± 1.88	0.033	2	0.861
	≥60	12	11.09 ± 1.26		3.67 ± 2.27		3	
Gender	F	9	12.03 ± 2.20	0.792	2.78 ± 1.86	0.421	1	0.476
	M	18	12.96 ± 1.73		3.44 ± 1.98		4	
Hemispheric dominance	Dominant	13	11.79 ± 2.33	0.693	2.26 ± 1.73	0.706	3	0.538
	Nondominant	14	12.36 ± 1.58		2.04 ± 2.05		2	
Infarction side	Left	10	10.49 ± 2.50	0.326	3.6 ± 2.07	0.581	3	0.675
	Right	17	12.18 ± 1.07		3 ± 1.87		2	
